# Galectin-3 and Autophagy in Renal Acute Tubular Necrosis

**DOI:** 10.3390/ijms25073604

**Published:** 2024-03-22

**Authors:** Suhail Al-Salam, Govindan S. Jagadeesh, Manjusha Sudhadevi, Javed Yasin

**Affiliations:** 1 Department of Pathology, College of Medicine and Health Sciences, United Arab Emirates University, Alain P.O. Box 15551, United Arab Emirates; 2 Department of Internal Medicine, College of Medicine and Health Sciences, United Arab Emirates University, Alain P.O. Box 15551, United Arab Emirates

**Keywords:** kidney, acute tubular necrosis, galectin-3, autophagy, macroautophagy, cell survival signals

## Abstract

Acute kidney injury (AKI) is a public health burden with increasing morbidity and mortality rates and health care costs. Acute tubular necrosis (ATN) is the most common cause of AKI. Cisplatin (CIS) is a platinum-based chemotherapeutic agent used in the treatment of a wide variety of malignancies such as lung, breast, ovary, testis, bladder, cervix, and head and neck cancers. Autophagy plays an important role in AKI. Galectin-3 (Gal-3) is significantly increased in renal tubules in AKI; however, its role in autophagy is not well understood. Male C57B6/J and B6.Cg-Lgals3 <^tm 1 Poi^>/J Gal-3 knockout (KO) mice were used to induce AKI using a CIS mouse model of ATN. Renal Gal-3 and autophagy proteins’ expression were measured using standard histologic, immunofluorescent, and enzyme-linked immunosorbent assay techniques. The data were presented as the mean ± S.E. Statistically significant differences (*p* < 0.05) were calculated between experimental groups and corresponding control groups by one-way analysis of variance. There was a significant increase in renal concentrations of Gal-3 in the Gal-3 wild-type CIS-treated mice when compared with sham control mice. There were significantly higher concentrations of renal LC3B, ATG13, Ulk-1, Beclin, ATG5, ATG12, ATG9A, and p-AMPK in the CIS-treated Gal-3 KO mice than in the Gal-3 wild-type CIS-treated mice. Further, there were significantly higher concentrations of mTOR, p- NF-κB, beta-catenin, and p62 in the kidneys of the Gal-3 wild-type CIS-treated mice than in the Gal-3 KO CIS-treated mice. Our findings affirm the connection between Gal-3 and autophagy, revealing its central role as a connector with prosurvival signaling proteins. Gal-3 plays a pivotal role in orchestrating cellular responses by interacting with prosurvival signal pathways and engaging with autophagy proteins. Notably, our observations highlight that the absence of Gal-3 can enhance autophagy in CIS-induced ATN.

## 1. Introduction

Acute kidney injury (AKI) frequently appears as a clinical manifestation in kidney care units, characterized by a sudden decline in renal function and concurrent disruptions in water, electrolyte, and protein homeostasis [1]. Individuals experiencing AKI face an elevated risk of morbidity and mortality. The primary culprits behind AKI include ischemia, nephrotoxins, and infections, each triggering inflammation. These conditions lead to direct damage to tubular cells, the initiation of oxidative stress, and dysfunction of the microvasculature endothelium [1]. Acute tubular necrosis (ATN) stands out as the predominant and shared histopathological alteration in all of these causative factors [1].

Cisplatin (CIS) is a platinum-based chemotherapeutic agent employed in treating various cancers, including lung, breast, ovary, testis, bladder, cervix, and head and neck malignancies [2]. However, its clinical utility is constrained by notable side effects, particularly nephrotoxicity. Presently, nearly a quarter of patients undergoing CIS treatment experience AKI, despite advancements in therapy [2]. Those who develop AKI face an elevated risk of mortality and a heightened likelihood of progressing to chronic kidney injury [2]. CIS-induced AKI encompasses diverse mechanisms, incorporating proximal tubular injury, vascular damage, inflammation, and oxidative stress. The primary manifestation of this injury is predominantly ATN, with a specific impact on the proximal tubules [3].

Autophagy serves as a dynamic intracellular balancing mechanism for energy and storage. This process facilitates cells’ recycling of endogenous materials and constructing essential macromolecules, and, thereby, maintaining cellular homeostasis while efficiently reusing energy [4,5]. Originally conceived as a catabolic process which provides nutrition and energy during cellular starvation, autophagy is a dynamic and intricate mechanism involving a series of cellular events. Autophagy is categorized into macroautophagy, microautophagy, and chaperone-mediated autophagy. Macroautophagy, referred to as autophagy, facilitates the degradation of large cytoplasmic materials by sequestering them in autophagosomes, which subsequently fuse with lysosomes for degradation. Microautophagy involves the direct sequestration of relatively small cytoplasmic substances into lysosomes through invagination of the lysosomal membrane. Chaperone-mediated autophagy represents a highly selective pathway wherein chaperone-HSC70 recognizes proteins containing the KFERQ motif [6].

The central events in autophagy (macroautophagy) involve the creation of autophagosomes and autolysosomes [6]. Various complexes, comprising autophagy-related proteins (ATG), collaborate with membrane transport components to induce autophagosome formation. The autophagic process encompasses five discernible stages: initiation (omegasome formation), vesicle nucleation (phagophore formation), vesicle elongation (autophagosome formation), vesicle fusion (autophagosome-lysosome fusion, resulting in autolysosome formation), and subsequent cargo degradation by lysosomal enzymes [6].

Recent evidence indicates that autophagy plays a pivotal role in both synthesis and degradation processes, engaging in intricate cross-interactions with apoptosis and cell cycle regulations [7]. Consequently, autophagy is implicated as a protective mechanism within living organisms and may exert an influential impact on the pathogenesis of various diseases [7]. The Adenosine Monophosphate-Activated Protein Kinase (AMPK) and the Mammalian Target of Rapamycin (mTOR) signaling pathway play crucial roles in tightly regulating autophagy [8]. The AMPK has the capability to inhibit TOR complex-1 (TORC1) activity, and, conversely, mTORC1 can reciprocally suppress the activation of AMPK [9]. It has been reported that mTOR inhibitors can activate autophagy [8].

Several reports indicate that autophagy exhibits renoprotective effects on proximal tubular cells during AKI [10]. Prior studies propose that autophagic responses to CIS treatment may confer protection to various cancer cells, potentially leading to CIS resistance [3]. It has been observed that CIS can activate autophagy in proximal tubules, resulting in substantial autophagosome formation and accumulation, which potentially serves a protective role [3]. Furthermore, mTOR inhibitors have been demonstrated to mitigate CIS-induced AKI in mice, improving their renal function through the up-regulation of autophagy [3]. Galectin-3 (Gal-3) is a distinctive chimera-like molecule classified within the lectin family that exhibits specific binding to N-acetyl-lactosamine-containing glycoproteins. Gal-3 assumes a significant role in numerous cellular physiological and pathological processes, encompassing cell proliferation, apoptosis, inflammation, and angiogenesis [11]. On reviewing the literature, few reports showed Gal-3 to be involved in autophagy [11,12,13]; however, its role and exact mechanism of action is still not known. Besides, nothing is known about its role in autophagy in AKI. Weng et al. have proposed a role for Gal-3 in the autophagic response [12]. A particular report has suggested that Gal-3 might mediate autophagy as a protective mechanism for cells against endomembrane damage linked to lysosomal dysfunction. Nevertheless, the precise mechanism remains not entirely understood [11].

Idikio et al. have demonstrated that Gal-3 and Beclin1 are implicated in two coordinated pathways of programmed cell death—apoptosis and autophagy [13].

In this study, we employed a murine model of AKI to explore the involvement of Gal-3 in autophagy during CIS-induced AKI. Our investigation involved the assessment of renal functions, including plasma urea and creatinine levels, kidney autophagy proteins, and cell survival markers (p-NFκ-B and beta-catenin). A standard enzyme-linked immunosorbent assay (ELISA), biochemical analyzer, and histologic and immunofluorescent procedures were utilized for our data collection. To test our hypothesis, we utilized both Gal-3 wild-type and Gal-3 KO mice.

## 2. Results

### 2.1. Cisplatin Induces AcuteKkidney Injury

#### 2.1.1. Plasma Urea

**Figure 1:**
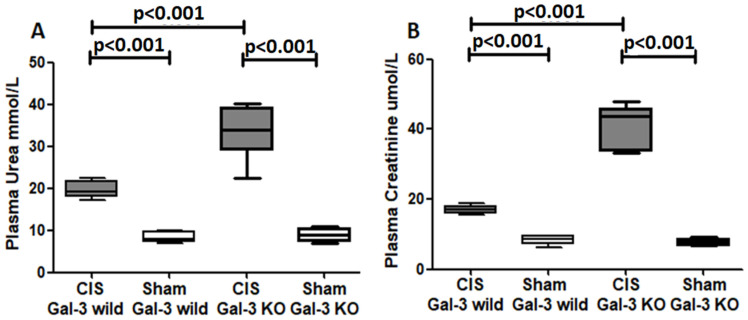
(**A**) The graph represents plasma urea concentrations following ATN in CIS-treated Gal-3 wild and KO mice compared to their sham controls. (**B**) The graph represents plasma creatinine concentrations following ATN in CIS-treated Gal-3 wild and KO mice compared to their sham controls.

A significant increase in plasma urea concentration was observed in the CIS-treated Gal-3 wild-type mice compared to Gal-3 wild-type sham control mice, indicating statistical significance (*p* < 0.001) (Figure 1A). Similarly, there was a significant elevation in the plasma urea concentration in the CIS-treated Gal-3 KO mice compared to Gal-3 KO control mice, also demonstrating statistical significance (*p* < 0.001) (Figure 1A). Furthermore, a significantly higher concentration of plasma urea was identified in the kidneys of the CIS-treated Gal-3 KO mice compared to the CIS-treated Gal-3 wild-type mice, showing statistical significance (*p* < 0.001) (Figure 1A). The results of multiple comparisons among the four experimental groups, utilizing one-way ANOVA followed by the Newman–Keuls post hoc test, were found to be statistically significant (*p* < 0.001).

#### 2.1.2. Plasma Creatinine

A significant increase in plasma creatinine concentration was noted in CIS-treated Gal-3 wild mice compared to Gal-3 wild-type sham control mice, indicating statistical significance (*p* < 0.001) (Figure 1B). Similarly, there was a significant elevation in plasma creatinine concentration in the CIS-treated Gal-3 KO mice compared to Gal-3 KO control mice, also demonstrating statistical significance (*p* < 0.001) (Figure 1B). Furthermore, a significantly higher concentration of plasma creatinine was identified in the kidneys of the CIS-treated Gal-3 KO mice compared to the CIS-treated Gal-3 wild-type mice, showing statistical significance (*p* < 0.001) (Figure 1B). The results of multiple comparisons among the four experimental groups, utilizing one-way ANOVA followed by the Newman–Keuls post hoc test, were found to be statistically significant (*p* < 0.001).

### 2.2. Cisplatin Induces Acute Tubular Necrosis

**Figure 2:**
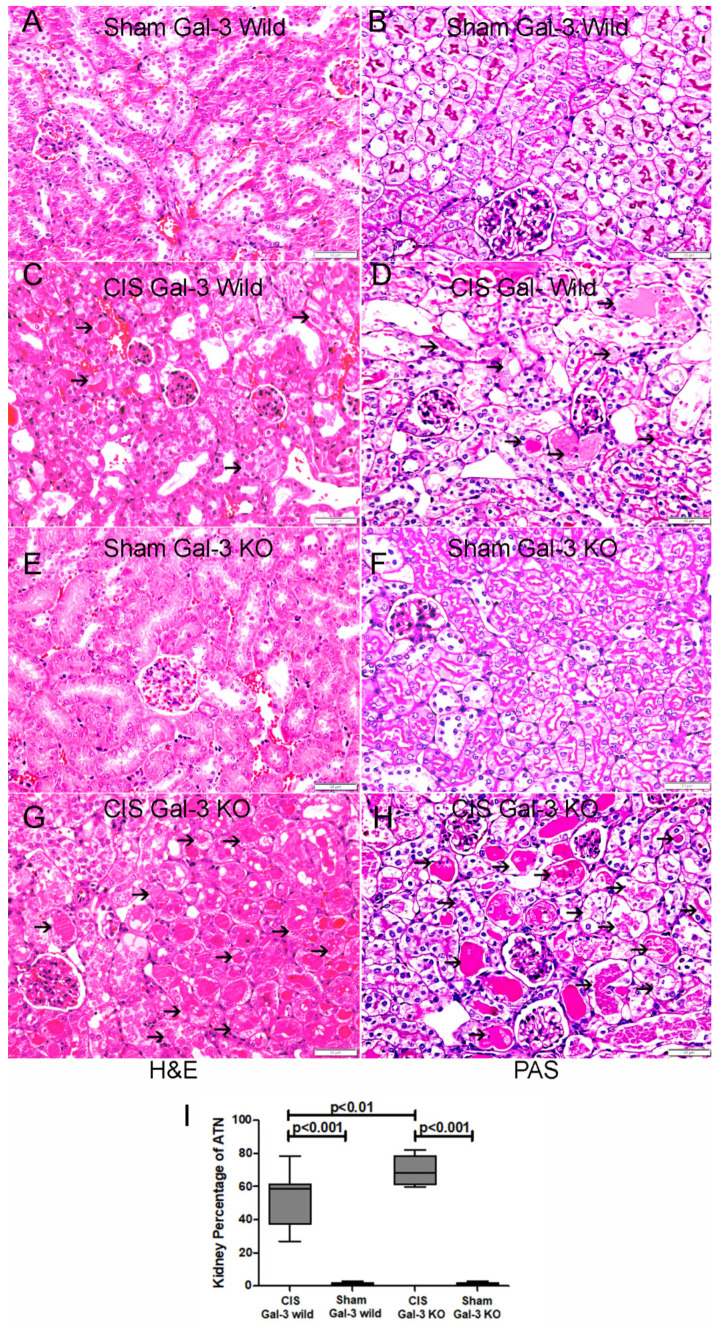
(**A**,**B**) Representative sections from the kidneys of saline-treated Gal-3 wild-type sham control mice, revealing unremarkable kidney parenchyma, as shown by H&E and PAS stains. (**C**,**D**) Representative section from the kidneys of CIS-treated Gal-3 wild-type mice, illustrating ATN characterized by tubular epithelial injury and intratubular secretion (thin arrow), as depicted by H&E and PAS stains. (**E**,**F**) Representative section from the kidneys of saline-treated Gal-3 KO sham control mice, displaying unremarkable kidney parenchyma, based on H&E and PAS stains. (**G**,**H**) Representative sections from the kidneys of CIS-treated Gal-3 knockout (KO) mice, exhibiting ATN with tubular epithelial injury and intratubular secretion (thin arrow), visualized through H&E and PAS stains. (**I**) The graph represents the percentage of ATN in kidneys of CIS-treated Gal-3 wild-type mice and CIS-treated Gal-3 KO mice compared to their corresponding sham controls; *p*-value < 0.05 is statistically significant.

The CIS-treated Gal-3 wild-type group showed ATN involving 51.98% ± 6.57% of renal parenchyma (Figure 2I) with athe loss of brush borders, swelling of tubular cells, intratubular falling cells, and intratubular secretions (Figure 2C,D).

The CIS-treated Gal-3 KO group showed ATN involving 69.66% ± 3.35% of renal parenchyma (Figure 2I) with the loss of brush borders, swelling of tubular cells, intratubular falling cells, intratubular secretions and mitosis (Figure 2G,H).

There was a significantly higher percentage of ATN in the kidneys in the CIS-treated GAL-3 KO mice than in the CIS-treated Gal-3 wild-type mice (*p* < 0.01) (Figure 2I).

### 2.3. Gal-3 Is Expressed in Acute Tubular Necrosis

**Figure 3:**
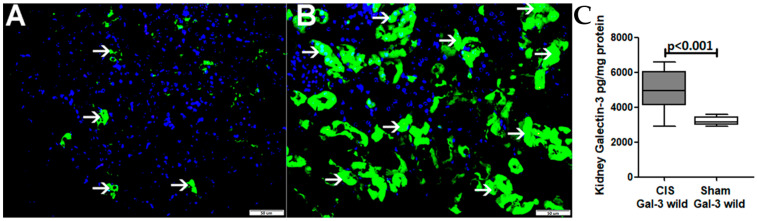
(**A**) Sections extracted from the kidneys of control mice treated with saline display minimal instances of tubules exhibiting cytoplasmic Gal-3 expression (thin arrow). (**B**) Sections from the kidneys of Gal-3 wild-type mice subjected to CIS treatment reveal a notable increase in cytoplasmic and nuclear Gal-3 expression in numerous tubules, particularly those associated with ATN (thin arrow). (**C**) The chart illustrates the concentrations of Gal-3 in the kidneys post ATN in Gal-3 wild-type mice treated with CIS, in comparison to their sham controls. A statistically significant difference is denoted by a *p*-value < 0.05.

There was a significant increase in the concentration of Gal-3 in the kidneys in the CIS-treated Gal-3 wild-type group when compared with the Gal-3 in those of the wild sham control group; *p* < 0.01 (Figure 3C).

Comparison between the experimental groups was done using Student’s *t*-test.

The immunofluorescent staining of representative kidney sections showed a significantly higher expression of Gal-3 in renal tubules affected by ATN in CIS Gal-3 wild-type mice (Figure 3B) when compared with those of Gal-3 wild-type sham control mice (Figure 3A).

### 2.4. Gal-3 Is Interacting with Autophagy Proteins

#### 2.4.1. Autophagy Flux

##### LC3B

**Figure 4:**
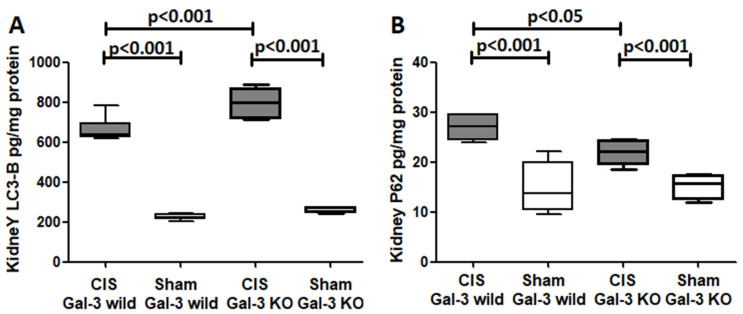
(**A**) The graph represents kidney LC3B concentrations following ATN in CIS-treated Gal-3 wild and KO mice compared to their sham controls. (**B**) The graph represents kidney p62 concentrations following ATN in CIS-treated Gal-3 wild and KO mice compared to their sham controls. *p*-value < 0.05 is statistically significant.

Autophagic flux is described as the rise in the lipidated 14 KDa LC3B form. In the kidneys of the Gal-3 wild-type mice treated with CIS, a notable increase in LC3B concentrations was observed in comparison to Gal-3 wild-type sham control mice (*p* < 0.001) (Figure 4A). Similarly, there was a significant rise in LC3B concentrations in the kidneys of the CIS-treated Gal-3 KO mice compared to those of Gal-3 KO sham control mice (*p* < 0.001) (Figure 4A). Interestingly, the concentrations of LC3B in the kidneys of the CIS-treated Gal-3 KO mice were significantly higher than in the CIS-treated Gal-3 wild-type mice (*p* < 0.001) (Figure 4A). The results of multiple comparisons among the four experimental groups using one-way ANOVA, followed by the Newman–Keuls post hoc test, were found to be statistically significant (*p* < 0.001).

**Figure 5:**
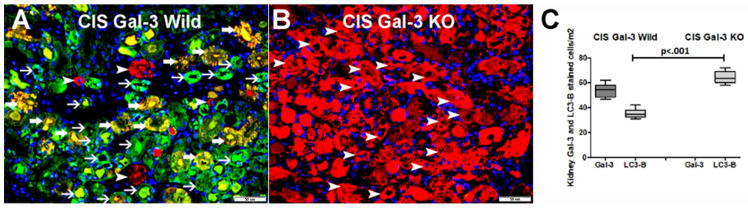
Double immunofluorescent staining of LC3B and Gal-3. (**A**) Representative section from kidneys of CIS-treated Gal-3 wild-type mice showing expression of Gal-3 (green) by tubular cells (thin arrow) and expression of LC3B (red) by tubular cells (arrowhead). Co-expression of LC3b and Gal-3 (yellow) was detected in some of the tubules (thick arrow). (**B**) Representative section from kidneys of CIS-treated Gal-3 KO mice showing clear higher expression of LC3B (red) by tubular cells (arrowhead) in the absence of Gal-3 expression when compared with A. (**C**). The graph represents morphometric analysis of kidney LC3B immunofluorescent staining following ATN in CIS-treated Gal-3 wild-type and CIS-treated KO mice in comparison to their sham controls. The graph illustrates a higher expression of LC3B in CIS-treated KO mice than other mice groups.

Double labeling immunofluorescent staining for LC3B and Gal-3 showed a significantly higher number of cells expressing LC3b in the kidneys of the CIS-treated GAL-3 KO mice than in the CIS-treated Gal-3 wild mice (*p* < 0.001) (Figure 5). Figure 5B clearly shows a significantly increased expression of LC3 B in renal tubules in the absence of Gal-3.

##### p62

**Figure 6:**
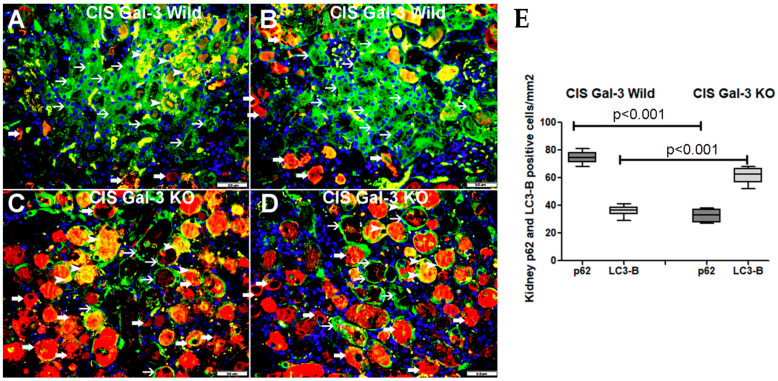
Immunofluorescent double labeling of LC3B and p62 showing autophagy flux in the presence and absence of Gal-3. (**A**,**B**) Representative sections from kidneys of CIS-treated Gal-3 wild-type mice showing expression of p62 (green) in the cytoplasm of tubular cells (thin arrow). There is cytoplasmic expression of LC3 B (red) in a number of tubular cells (thick arrow). There is co-expression of p62 and LC3B (yellow) in the cytoplasm of some tubular cells’ lysosomes/vacuoles (arrowhead). (**C**,**D**) Representative sections from kidneys of CIS-treated Gal-3 KO mice showing lower expression of p62 (green) in the cytoplasm of tubular cells (thin arrow). There is higher and diffuse cytoplasmic expression of LC3 B (red) in tubular cells (thick arrow). There is co-expression of p62 and LC3b (yellow) in the cytoplasm of some tubular cells’ lysosomes/vacuoles (arrowhead). (**E**) The graph represents morphometric analysis of kidney p62 and LC3B immunofluorescent staining following ATN in CIS-treated Gal-3 wild-type and CIS-treated KO mice in comparison to their sham controls. The graph illustrates higher expression of LC3B in CIS-treated KO mice than in CIS-treated Gal-3 wild mice as well as higher expression of p62 in CIS-treated Gal-3 wild mice than in CIS-treated KO mice.

A marked increase in p62 concentrations was observed in the kidneys of the CIS-treated Gal-3 wild-type mice in comparison to Gal-3 wild-type sham control mice, indicating statistical significance (*p* < 0.001) (Figure 4B). Similarly, there was a notable elevation in p62 concentrations in the kidneys of the CIS-treated Gal-3 KO mice when compared with Gal-3 KO control mice, also demonstrating statistical significance (*p* < 0.001) (Figure 4B). Moreover, a significantly higher concentration of p62 was found in the kidneys of the CIS-treated Gal-3 wild-type mice than in the CIS-treated Gal-3 KO mice, with statistical significance (*p* < 0.05) (Figure 4B). Immunofluorescent staining for p62 revealed a significantly higher number of cells expressing p62 in the kidneys of the CIS-treated Gal-3 wild-type mice then in the CIS-treated Gal-3 KO mice (Figure 6). The results of multiple comparisons among the four experimental groups, utilizing one-way ANOVA followed by the Newman–Keuls post hoc test, were found to be statistically significant (*p* < 0.001).

##### Autophagy FLUX

The ELISA measurements of the LC3B and p62 concentrations exhibited a significant increase in kidney LC3B concentrations in both the Gal-3 wild-type and Gal-3 KO mice following CIS-induced AKI. This implies an augmentation in autophagy flux within tubular cells in response to CIS insult to renal tubular cells. Given that p62 is actively involved in autophagy flux, a higher autophagy flux is generally associated with lower levels of p62. Consequently, we observed a reverse relationship between LC3B and p62. In CIS-treated Gal-3 KO mice, a higher concentration of LC3B was identified, accompanied by a lower concentration of p62 in tubular cells compared to the CIS-treated Gal-3 wild-type mice. This observation suggests that the loss of Gal-3 is linked to an increase in autophagic flux, emphasizing the potentially significant role of Gal-3 in autophagy (Figure 4, Figure 5 and Figure 6).

##### p-AMPK

**Figure 7:**
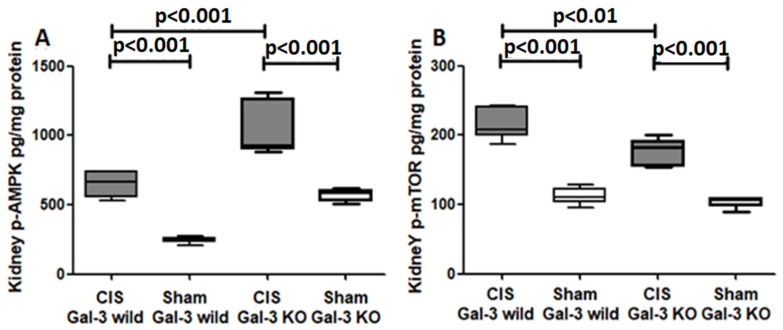
(**A**) The chart depicts kidney p-AMPK concentrations after ATN in CIS-treated Gal-3 wild-type and KO mice, relative to their respective sham controls. (**B**) The graph illustrates kidney p-mTOR concentrations post ATN in CIS-treated Gal-3 wild-type and KO mice compared to their sham controls. A statistically significant difference is indicated by a *p*-value < 0.05.

**Figure 8:**
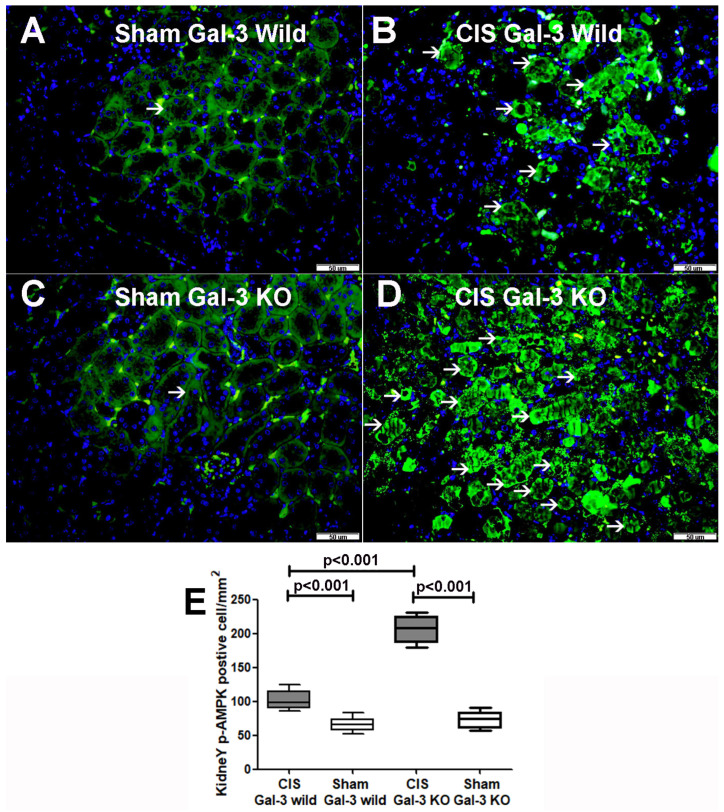
(**A**). Sections extracted from the kidneys of saline-treated Gal-3 wild-type sham control mice exhibit low cytoplasmic expression of p-AMPK by tubular cells (thin arrow). (**B**) Sections from kidneys of CIS-treated Gal-3 wild-type mice display elevated expression of p-AMPK in the cytoplasm of tubular cells (thin arrow) compared to panel A. (**C**) Sections from kidneys of saline-treated Gal-3 KO sham control mice show minimal cytoplasmic expression of p-AMPK by tubular cells (thin arrow). (**D**) Sections from kidneys of CIS-treated Gal-3 KO mice reveal increased expression of p-AMPK in the cytoplasm of tubular cells (thin arrow) compared to panels (**A**–**C**,**E**). The graph represents morphometric analysis of kidney p-AMPK immunofluorescent staining following ATN in CIS-treated Gal-3 wild-type and CIS-treated KO mice in comparison to their sham controls. The graph illustrates higher expression of p-AMPK in CIS-treated KO mice than in other mice groups.

A noteworthy increase in p-AMPK concentrations was observed in the kidneys of CIS-treated Gal-3 wild-type mice when compared to Gal-3 wild-type sham control mice, indicating statistical significance (*p* < 0.001) (Figure 7A). Similarly, there was a significant elevation in p-AMPK concentrations in the kidneys of the CIS-treated Gal-3 KO mice when compared with Gal-3 KO sham control mice, also demonstrating statistical significance (*p* < 0.001) (Figure 7A). Moreover, a significantly higher concentration of p-AMPK was identified in the kidneys of the CIS-treated Gal-3 KO mice compared to the CIS-treated Gal-3 wild-type mice, with statistical significance (*p* < 0.001) (Figure 7A). Immunofluorescent staining for p-AMPK revealed a significantly higher number of cells expressing p-AMPK in the kidneys of the CIS-treated Gal-3 KO mice (Figure 8D,E) than in the CIS-treated Gal-3 wild-type mice (*p* < 0.001) (Figure 8B,E). The results of multiple comparisons among the four experimental groups, utilizing one-way ANOVA followed by the Newman–Keuls post hoc test, were found to be statistically significant (*p* < 0.001). It is worth noting that AMPK serves as a crucial energy sensor in maintaining cellular energy homeostasis, triggering the autophagy process in various situations and potentially being linked with different stages of autophagy.

##### p-mTOR

**Figure 9:**
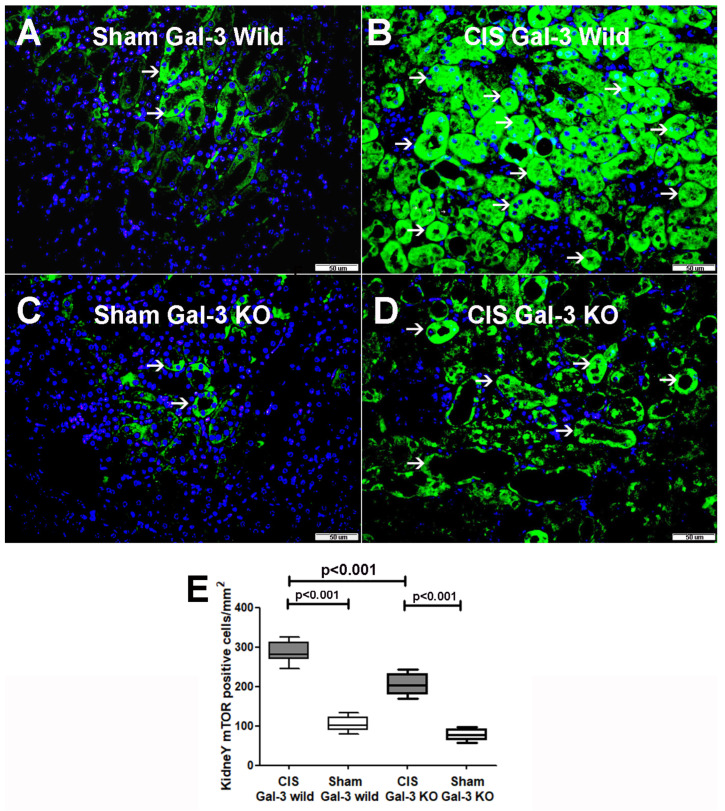
(**A**) Representative sections from kidneys of saline-treated Gal-3 wild-type sham control mice showing focal cytoplasmic expression of p-mTOR by tubular cells (thin arrow). (**B**) Representative sections from kidneys of CIS-treated Gal-3 wild-type mice showing higher expression of p-mTOR in the cytoplasm of tubular cells (thin arrow) than (**A**). (**C**) Representative sections from kidneys of saline-treated Gal-3 KO sham control mice showing focal cytoplasmic expression of p-mTOR by tubular cells (thin arrow). (**D**) Representative sections from kidneys of CIS-treated Gal-3 KO mice showing higher expression of p-mTOR in the cytoplasm of tubular cells (thin arrow) than (**C**). (**E**) The graph represents morphometric analysis of kidney p-mTOR immunofluorescent staining following ATN in CIS-treated Gal-3 wild-type and CIS-treated KO mice compared to their sham controls, showing higher expression of p-mTOR in CIS-treated Gal-3-type mice (**A**) than (**B**–**D**).

There was a notable increase in p-mTOR concentrations in the kidneys of the CIS-treated Gal-3 wild-type mice compared to Gal-3 wild-type sham control mice, signifying statistical significance (*p* < 0.001) (Figure 7B). Similarly, a significant elevation in p-mTOR concentrations was observed in the kidneys of the CIS-treated Gal-3 KO mice compared to Gal-3 KO sham control mice, demonstrating statistical significance (*p* < 0.001) (Figure 7B). Moreover, there were significantly higher concentrations of p-mTOR in the kidneys of the CIS-treated Gal-3 wild-type mice than in the CIS-treated Gal-3 KO mice, with statistical significance (*p* < 0.01) (Figure 7B). Immunofluorescent staining for p-mTOR revealed a significantly higher number of cells expressing mTOR in the kidneys of the CIS-treated Gal-3 wild-type mice (Figure 9B,E) than in the CIS-treated Gal-3 KO mice (*p* < 0.001) (Figure 9D,E). The results of multiple comparisons among the four experimental groups, using one-way ANOVA followed by the Newman–Keuls post hoc test, were found to be statistically significant (*p* < 0.001). It is worth noting that mTOR is an anti-autophagic protein, and its activation inhibits autophagy [3].

##### ATG13

**Figure 10:**
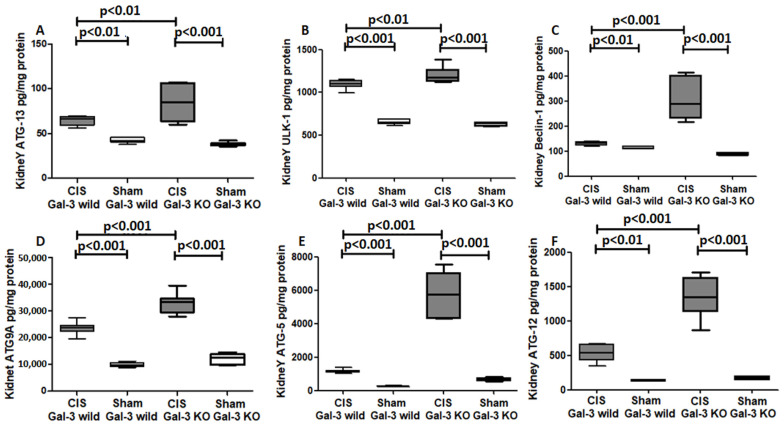
(**A**) The graph represents kidney ATG13 concentrations following ATN in CIS-treated Gal-3 wild and KO mice compared to their sham controls. (**B**) The graph represents kidney Alk-1 concentrations following ATN in CIS-treated Gal-3 wild and KO mice compared to their sham controls. (**C**) The graph represents kidney Beclin-1 concentrations following ATN in CIS-treated Gal-3 wild and KO mice compared to their sham controls. (**D**) The graph represents kidney ATG9A concentrations following ATN in CIS-treated Gal-3 wild and KO mice compared to their sham controls. (**E**) The graph represents kidney ATG5 concentrations following ATN in CIS-treated Gal-3 wild and KO mice compared to their sham controls. (**F**) The graph represents kidney ATG12 concentrations following ATN in CIS-treated Gal-3 wild and KO mice compared to their sham controls.

A significant increase in ATG13 concentrations was observed in the kidneys of the CIS-treated Gal-3 wild-type mice in comparison to Gal-3 wild-type sham control mice, indicating statistical significance (*p* < 0.001) (Figure 10A). Similarly, there was a significant elevation in ATG13 concentrations in the kidneys of the CIS-treated Gal-3 KO mice compared to Gal-3 KO sham control mice, also demonstrating statistical significance (*p* < 0.001) (Figure 10A). Furthermore, significantly higher concentrations of ATG13 were identified in the kidneys of the CIS-treated Gal-3 KO mice compared to the CIS-treated Gal-3 wild-type mice, with statistical significance (*p* < 0.001) (Figure 10A). The results of multiple comparisons among the four experimental groups, utilizing one-way ANOVA followed by the Newman–Keuls post hoc test, were found to be statistically significant (*p* < 0.001).

##### Ulk-1 (Atg1)

A significant increase in Ulk-1 concentrations was noted in the kidneys of the CIS-treated Gal-3 wild-type mice compared to Gal-3 wild-type sham control mice, demonstrating statistical significance (*p* < 0.01) (Figure 10B). Similarly, there was a significant elevation in Ulk-1 concentrations in the kidneys of the CIS-treated Gal-3 KO mice compared to Gal-3 KO sham control mice, also displaying statistical significance (*p* < 0.001) (Figure 10B). Furthermore, significantly higher concentrations of Ulk-1 were identified in the kidneys of the CIS-treated Gal-3 KO mice compared to the CIS-treated Gal-3 wild-type mice, with statistical significance (*p* < 0.001) (Figure 10B). The results of multiple comparisons among the four experimental groups, utilizing one-way ANOVA followed by the Newman–Keuls post hoc test, were found to be statistically significant (*p* < 0.001).

##### Beclin-1

A significant increase in Beclin-1 concentrations was observed in the kidneys of CIS-treated Gal-3 wild-type mice compared to Gal-3 wild-type sham control mice, showing statistical significance (*p* < 0.01) (Figure 10C). Similarly, there was a significant elevation in Beclin-1 concentrations in the kidneys of the CIS-treated Gal-3 KO mice compared to Gal-3 KO sham control mice, also demonstrating statistical significance (*p* < 0.001) (Figure 10C). Furthermore, significantly higher concentrations of Beclin-1 were identified in the kidneys of the CIS-treated Gal-3 KO mice compared to the CIS-treated Gal-3 wild-type mice, with statistical significance (*p* < 0.001) (Figure 10C). The results of multiple comparisons among the four experimental groups, utilizing one-way ANOVA followed by the Newman–Keuls post hoc test, were found to be statistically significant (*p* < 0.001).

##### ATG9A

A significant increase in ATG9A concentrations was evident in the kidneys of the CIS-treated Gal-3 wild-type mice when compared with Gal-3 wild-type sham control mice, signifying statistical significance (*p* < 0.001) (Figure 10D). Similarly, there was a significant elevation in ATG9A concentrations in the kidneys of the CIS-treated Gal-3 KO mice compared to Gal-3 KO sham control mice, also demonstrating statistical significance (*p* < 0.001) (Figure 10D). Moreover, significantly higher concentrations of ATG9A were identified in the kidneys of the CIS-treated Gal-3 KO mice compared to the CIS-treated Gal-3 wild-type mice, with statistical significance (*p* < 0.001) (Figure 10D). The results of multiple comparisons among the four experimental groups, utilizing one-way ANOVA followed by the Newman–Keuls post hoc test, were found to be statistically significant (*p* < 0.001).

##### ATG5

A significant increase in ATG5 concentrations was observed in the kidneys of the CIS-treated Gal-3 wild-type mice compared to Gal-3 wild-type sham control mice, indicating statistical significance (*p* < 0.001) (Figure 10E). Similarly, there was a significant elevation in ATG5 concentrations in the kidneys of the CIS-treated Gal-3 KO mice compared to Gal-3 KO sham control mice, also demonstrating statistical significance (*p* < 0.001) (Figure 10E). Furthermore, significantly higher concentrations of ATG5 were identified in the kidneys of the CIS-treated Gal-3 KO mice compared to the CIS-treated Gal-3 wild-type mice, with statistical significance (*p* < 0.001) (Figure 10E). The results of multiple comparisons among the four experimental groups, utilizing one-way ANOVA followed by the Newman–Keuls post hoc test, were found to be statistically significant (*p* < 0.001).

##### ATG 12

A significant increase in ATG12 concentrations was observed in the kidneys of the CIS-treated Gal-3 wild-type mice compared to Gal-3 wild-type sham control mice, signifying statistical significance (*p* < 0.001) (Figure 10F). Similarly, there was a significant elevation in ATG12 concentrations in the kidneys of the CIS-treated Gal-3 KO mice compared to Gal-3 KO sham control mice, also demonstrating statistical significance (*p* < 0.001) (Figure 10F). Moreover, significantly higher concentrations of ATG12 were identified in the kidneys of the CIS-treated Gal-3 KO mice compared to the CIS-treated Gal-3 wild-type mice, with statistical significance (*p* < 0.001) (Figure 10F). The results of multiple comparisons among the four experimental groups, utilizing one-way ANOVA followed by the Newman–Keuls post hoc test, were found to be statistically significant (*p* < 0.001).

### 2.5. GAL-3 Is Interacting with Prosurvival Signals

#### 2.5.1. Phospho-NF-κB

**Figure 11:**
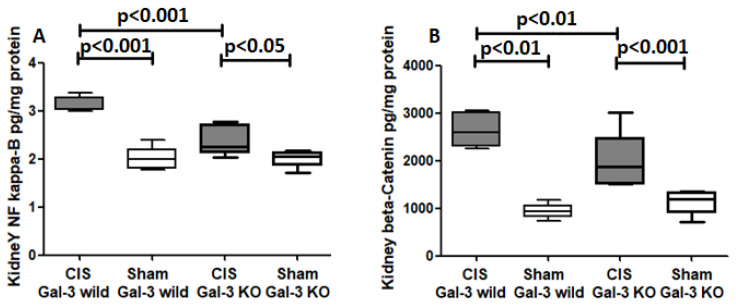
(**A**) The graph represents kidney phospho-NF-κB concentrations following ATN in CIS-treated Gal-3 wild and KO mice compared to their sham controls. (**B**) The graph represents kidney beta-catenin concentrations following ATN in CIS-treated Gal-3 wild and KO mice compared to their sham controls.

**Figure 12:**
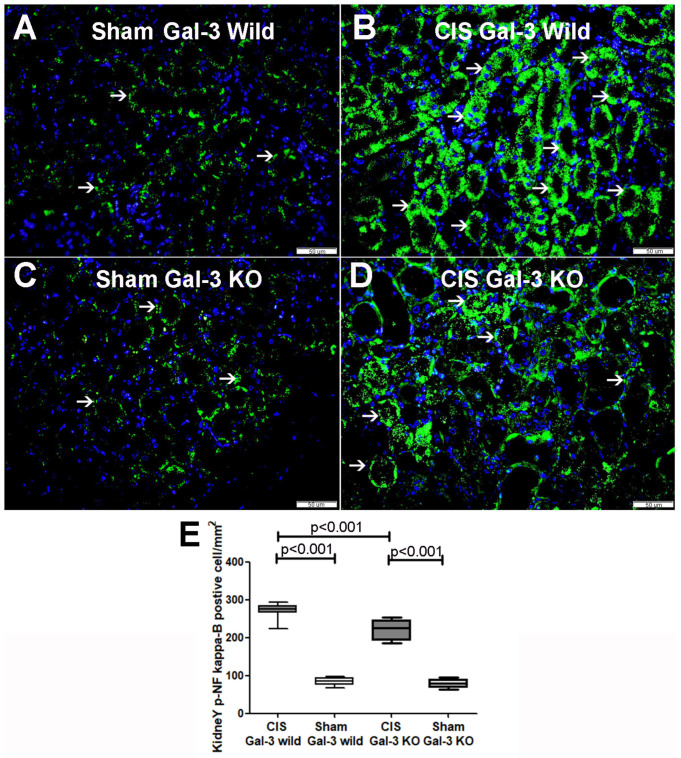
(**A**) Representative sections from kidneys of saline-treated Gal-3 wild-type sham control mice showing focal cytoplasmic expression of phospho-NF-κB by tubular cells (thin arrow). (**B**) Representative sections from kidneys of CIS-treated Gal-3 wild-type mice showing high expression of phospho-NF-κB in the cytoplasm of tubular cells (thin arrow). (**C**) Representative sections from kidneys of saline-treated Gal-3 KO sham control mice showing focal cytoplasmic expression of phospho-NF-κB by tubular cells (thin arrow). (**D**) Representative sections from kidneys of CIS-treated Gal-3 KO mice showing high expression of phospho-NF-κB in the cytoplasm of tubular cells (thin arrow). (**E**) The graph represents morphometric analysis of kidney phospho-NF-κB immunofluorescent staining following ATN in CIS-treated Gal-3 wild-type and CIS-treated KO mice compared to their sham controls, showing higher expression of phospho-NF-κB in tubular cells in CIS-treated Gal-3 wild-type mice (**A**) than (**B**–**D**).

A significant increase in phospho-NF-κB concentrations were observed in the kidneys of the CIS-treated Gal-3 wild-type mice compared to Gal-3 wild-type sham control mice, demonstrating statistical significance (*p* < 0.001) (Figure 11A). Similarly, there was a significant elevation in phospho-NF-κB concentrations in the kidneys of the CIS-treated Gal-3 KO mice compared to Gal-3 KO sham control mice, with statistical significance (*p* < 0.05) (Figure 11A). Furthermore, significantly higher concentrations of phospho-NF-κB were identified in the kidneys of the CIS-treated Gal-3 wild-type mice compared to the CIS-treated Gal-3 KO mice, showing statistical significance (*p* < 0.001) (Figure 11A). Immunofluorescent staining of phospho-NF-κB revealed significantly higher expressions in the renal tubules of the CIS-treated Gal-3 wild mice compared to Gal-3 wild-type sham control mice (*p* < 0.001) (Figure 12A,B,E). Similarly, there was significantly higher expressions of phospho-NF-κB in the renal tubules of the CIS-treated Gal-3 KO mice compared to Gal-3 KO sham control mice (*p* < 0.001) (Figure 12C–E). Moreover, there were significantly higher concentrations of phospho-NF-κB in the kidneys of the CIS-treated Gal-3 wild-type mice compared to the CIS-treated Gal-3 KO mice, with statistical significance (*p* < 0.01) (Figure 12B,D,E). The results of multiple comparisons among the four experimental groups, utilizing one-way ANOVA followed by the Newman–Keuls post hoc test, were found to be statistically significant (*p* < 0.001).

#### 2.5.2. Beta-Catenin

**Figure 13:**
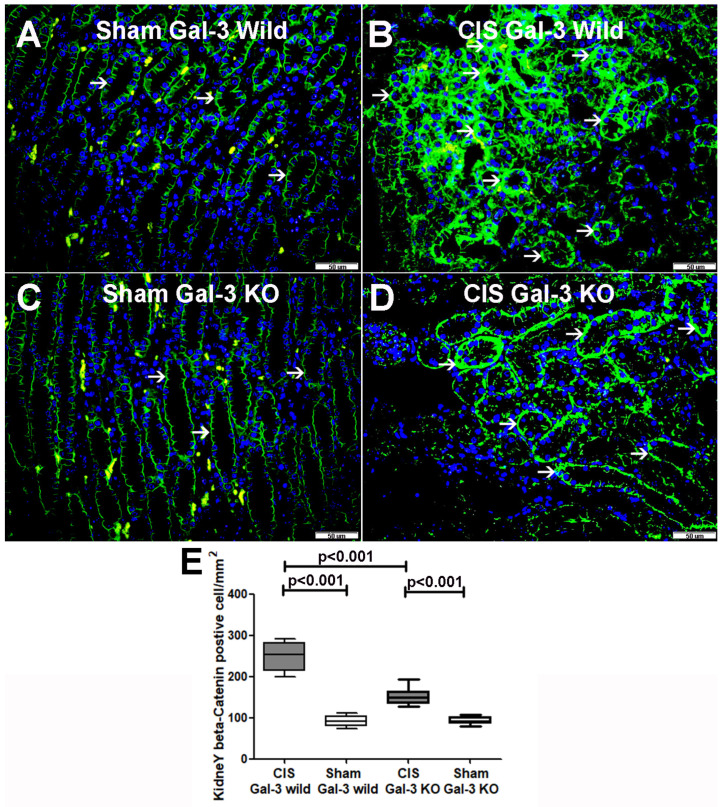
(**A**) Representative sections from kidneys of saline-treated Gal-3 wild-type sham control mice showing focal membranous expression of beta-catenin by tubular cells (thin arrow). (**B**) Representative sections from kidneys of CIS-treated Gal-3 wild-type mice showing higher cytoplasmic expression of beta-catenin in tubular cells (thin arrow) than (**A**). (**C**) Representative sections from kidneys of saline Gal-3 KO sham control mice showing focal membranous expression of beta-catenin by tubular cells (thin arrow). (**D**) Representative sections from kidneys of CIS Gal-3 KO mice showing higher expression of beta-catenin in the cytoplasm of tubular cells (thin arrow) than (**C**). (**E**) The graph represents morphometric analysis of kidney beta-catenin immunofluorescent staining following ATN in CIS-treated Gal-3 wild-type and CIS-treated KO mice compared to their sham controls, showing higher expression of beta-catenin in tubular cells in CIS-treated Gal-3 wild-type mice (**A**) than (**B**–**D**).

A significant increase in beta-catenin concentrations was noted in the kidneys of the CIS-treated Gal-3 wild-type mice compared to Gal-3 wild-type sham control mice, indicating statistical significance (*p* < 0.01) (Figure 11B). Similarly, there was a significant elevation in beta-catenin concentrations in the kidneys of the CIS-treated Gal-3 KO mice compared to Gal-3 KO sham control mice, demonstrating statistical significance (*p* < 0.001) (Figure 11B). Furthermore, significantly higher concentrations of beta-catenin were identified in the kidneys of the CIS-treated Gal-3 wild-type mice compared to the CIS-treated Gal-3 KO mice, showing statistical significance (*p* < 0.01) (Figure 11B). Immunofluorescent staining of beta-catenin revealed significantly higher expressions in the renal tubules of the CIS-treated Gal-3 wild mice compared to Gal-3 wild-type sham control mice (*p* < 0.001) (Figure 13A,B,E). Similarly, there were significantly higher expressions of beta-catenin in the renal tubules of the CIS-treated Gal-3 KO mice compared to Gal-3 KO sham control mice (*p* < 0.001) (Figure 13C,D). Moreover, there were significantly higher concentrations of beta-catenin in the renal tubules of the CIS-treated Gal-3 wild-type mice compared to the CIS-treated Gal-3 KO mice, with statistical significance (*p* < 0.01) (Figure 13B,D,E). The results of multiple comparisons among the four experimental groups, utilizing one-way ANOVA followed by the Newman–Keuls post hoc test, were found to be statistically significant (*p* < 0.001).

## 3. Discussion

Acute kidney injury represents a prevalent global health challenge, with a significantly high number of new cases diagnosed annually, nearly 85% of which occur in developing countries [14]. This burden is escalating, particularly in developing nations [15]. On a global scale, AKI contributes to 1.7 million deaths each year, with almost 1.4 million of these occurring in low- and middle-income countries [15]. AKI is often preventable and treatable, with few, if any, cases resulting in long-term health consequences. Identifying the key mechanisms and participants in AKI’s development holds the potential to enhance patient care, thereby minimizing mortality and morbidity [15]. Autophagy, a lysosomal degradation pathway, serves as a mechanism for clearing defective or damaged cytoplasmic structures, misfolded proteins, and intracellular debris [16]. The goal is to enable their reuse, aiming to restore cellular homeostasis [16]. In the kidney, the maintenance of a normal level of autophagy is crucial for preserving renal cell homeostasis and function under ordinary physiological conditions [16]. Understanding the intricate relationship between autophagy and AKI could offer valuable insights for improving patient outcomes and managing this global health issue more effectively. Autophagy is triggered in response to cellular stress, particularly when the kidney is diseased or subjected to insults or toxins, such as CIS [17]. Autophagy is generally recognized as a crucial process for promoting cell survival and providing protection against acute nephrotoxicity induced by CIS [18]. Reports suggest that autophagy plays a protective role in cases of AKI induced by CIS [19]. Additionally, there is evidence indicating that autophagy can induce resistance to CIS treatment in cancer cells [20]. The intricate relationship between autophagy, CIS treatment, and renal health underscores the need for a comprehensive understanding to potentially enhance therapeutic strategies and mitigate adverse effects. Our findings provide valuable insights into the role of Gal-3 in ATN and its association with autophagy. The increased concentrations of Gal-3 in kidneys affected by AKI and its significantly higher expression in renal tubules affected by ATN in CIS-treated Gal-3 wild-type mice compared to controls suggest Gal-3 as a potential player in ATN.

The observation of significantly higher plasma concentrations in CIS-treated mice compared to saline-treated mice can be used to confirm AKI. The use of Gal-3 KO mice to investigate the mechanistic role of Gal-3 in autophagy during ATN is a pertinent approach. The observation of a significantly higher fraction of tubules affected by ATN in CIS-treated Gal-3 KO mice than in CIS-treated Gal-3 wild-type mice indicates that the absence of Gal-3 is associated with more tubular damage [21]. This is supported by the higher levels of plasma creatinine and urea in CIS-treated Gal-3 KO than in CIS-treated Gal-3 wild-type mice.

The alterations in autophagic markers, such as LC3B and p62, further shed light on the role of Gal-3 in modulating autophagy. The significant increase in LC3B concentrations in the kidneys of CIS-treated mice, particularly in Gal-3 KO mice, suggests a higher autophagic flux. LC3B’s central role in autophagy, involving vesicle elongation, maturation, the fusion of autophagosomes to lysosomes, and cargo recognition, supports the interpretation of increased autophagy flux [22,23]. Additionally, the significantly lower concentrations of p62 in CIS-treated Gal-3 KO mice compared to CIS-treated Gal-3 wild-type mice align with the notion of increased autophagic flux. p62 is typically degraded during autophagy, and its reduced levels in Gal-3 KO mice indicate enhanced autophagic turnover. The current study provides valuable contributions to understanding the interplay between Gal-3, autophagy, and renal injury. The evidence presented herein suggests that Gal-3 may play a role in modulating autophagy and influencing the extent of tubular damage in the context of AKI induced by CIS.

p62, also known as sequestosome-1 (SQSTM1), acts as a crucial adaptor protein in autophagy by delivering ubiquitinated cargoes to autophagosomes for degradation. As a result, the activation of autophagy leads to a reduction in p62 expression [24]. Due to its role as an autophagic substrate, p62 is frequently employed as a reliable indicator or predictor of autophagic flux [25]. Monitoring changes in p62 levels provides valuable insights into the dynamics of autophagy, where a decrease in p62 expression is indicative of increased autophagic activity and successful cargo degradation within the autophagic process [26].

Therefore, the observed lower concentrations of p62 in CIS-treated Gal-3 KO mice compared to CIS-treated Gal-3 wild-type mice in our study align with the notion of heightened autophagic flux in the absence of Gal-3.

Our findings regarding the significant increase in the concentrations of various autophagy proteins, including Ulk-1, ATG13, Beclin-1, ATG5, ATG12, and ATG9A, in kidneys affected by CIS-induced ATN compared to sham controls indicate an activation of autophagy in response to CIS insult. This aligns with the notion that autophagy is often induced under stress conditions, such as nephrotoxic insults. Moreover, the observed significantly higher concentrations of these autophagy proteins in CIS-treated Gal-3 KO mice compared to those in CIS-treated Gal-3 wild-type mice suggest that the absence of the Gal-3 gene can enhance autophagy. Ulk-1 kinase complex is recognized as functioning in the initiation stage of the autophagy pathway, while ATG13 plays a crucial role in governing the formation of the Ulk-1(Atg1) complex in a dephosphorylation-dependent manner [27], whereas Beclin-1 is capable of intervening at every major step in autophagic pathways, from phagophore and autophagosome formation to autophagosome/endosome maturation [28]. ATG9A, a multispanning membrane protein residing in Golgi membranes and endosomes, traffics to sites of autophagosome formation and supplies proteins and lipids to the autophagosome during the activation of autophagy [29]. Remarkably, ATG5 forms a complex with ATG12 and ATG16, subsequently conjugating with LC3B, and this complex is deposited on the membrane of the autophagosome [30].

Our finding of increased levels of autophagy-related proteins in Gal-3 KO mice provides further support to the idea that Gal-3 may act as a negative regulator of autophagy in the context of CIS-induced AKI. 

Our findings regarding the significantly higher concentrations of kidney-phosphorylated AMP-activated protein kinase (p-AMPK) in CIS-treated Gal-3 KO mice compared to CIS-treated Gal-3 wild-type mice are noteworthy. This observation suggests that knocking out the Gal-3 gene increases autophagy, and the involvement of p-AMPK supports this idea. AMPK is known to play a key role in regulating various stages of autophagy. By directly phosphorylating Ulk-1 at several sites, including Ser467, Ser555, Thr574, and Ser637, AMPK enhances Ulk-1 activity [31]. This, in turn, promotes the transcription and recruitment of autophagy-relevant proteins (ATG proteins) to the membrane domains, thereby activating autophagy at the initiation stage, leading towards nucleation, autophagosome formation, and ultimately autolysosome formation [31,32].

The higher concentrations of p-AMPK in Gal-3 KO mice further suggest that Gal-3 may act as a negative regulator of AMPK-mediated autophagy.

The observed lower concentrations of kidney p-mTOR (phosphorylated mammalian target of rapamycin) in CIS-treated Gal-3 KO mice compared to those in CIS-treated Gal-3 wild-type mice align with the well-established role of mTOR in suppressing autophagy. mTOR achieves this suppression by phosphorylating Ulk-1 and ATG13, thereby inhibiting the formation of the Ulk-1–Ulk-2 complex and preventing the initiation of autophagy [16]. The intricate relationship between AMPK, mTOR, and autophagy is well-documented. p-AMPK can directly induce autophagy by phosphorylating Ulk-1, and it can also inhibit mTOR through phosphorylation, indirectly promoting autophagy [32]. In the context of our study, the higher concentrations of p-AMPK and lower concentrations of p-mTOR in Gal-3 KO mice suggest that the loss of the Gal-3 gene may increase autophagy through the upregulation of p-AMPK and the inhibition of mTOR. The reciprocal regulation between AMPK and mTOR, where AMPK inhibits TOR complex-1 (TORC1) activity, and TORC1 reciprocally suppresses AMPK activation, further supports the idea that the loss of Gal-3 may disrupt this balance in favor of increased autophagy [33]. Our findings provide valuable insights into the molecular mechanisms through which Gal-3 may influence autophagy, involving the intricate interplay between AMPK and mTOR. Understanding these regulatory pathways could contribute to the development of targeted interventions for conditions associated with dysregulated autophagy, such as kidney injury induced by CIS.

The observed increase in kidney concentrations of phosphorylated NF-κB (p-NF-κB) and beta-catenin, in both CIS-treated Gal-3 wild-type and KO mice, with significantly higher levels in Gal-3 wild-type mice compared to Gal-3 KO mice, supports the idea that the absence of Gal-3 is associated with lower concentrations of these signaling molecules.

NF-κB and beta-catenin are key signaling proteins involved in various cellular processes, including inflammation, cell survival, and cell proliferation. The modulation of these signaling pathways could have implications for the cellular responses to stress and injury, and the overall outcome of kidney injury, induced by CIS [34,35,36,37].

The differences in the concentrations of these signaling molecules between Gal-3 wild-type and KO mice suggest that Gal-3 may play a regulatory role in these pathways. Further investigations into the specific mechanisms through which Gal-3 influences NF-κB and beta-catenin signaling could provide deeper insights into the molecular events underlying kidney injury and may offer potential therapeutic targets for intervention.

The findings from Wu et al. [34], indicating a significant increase in p-NF-κB in kidneys with ATN, align with our observations of increased p-NF-κB levels in CIS-treated Gal-3 wild-type and KO mice. This supports the notion that NF-κB activation is associated with kidney injury, particularly in the context of ATN [35]. Furthermore, the findings from Salminen et al. add a layer of complexity to the relationship between NF-κB and autophagy. The NF-κB signaling pathway is known to activate mTOR kinase, which is a major inhibitor of autophagy. Therefore, the suppression of NF-κB could potentially relieve the inhibitory effect on mTOR and, consequently, increases autophagy. This information resonates with our observations of higher p-NF-κB levels in Gal-3 wild-type mice compared to those in Gal-3 KO mice, coupled with our earlier findings of higher autophagy in Gal-3 KO mice. The intricate cross-talk between NF-κB, mTOR, and autophagy adds another layer to the complex regulatory network that Gal-3 may be involved in. 

Our observation of NF-κB signaling increasing the expression of NOD-like receptors with purine domain (NLRP) receptors is interesting in the context of autophagy [35]. The interaction between NLRP receptors and Beclin-1, leading to the inhibition of autophagy, adds another layer to the regulatory mechanisms involved in cellular responses to proinflammatory conditions [35]. This interplay between NF-κB, NLRP receptors, and Beclin-1 further highlights the complexity of the regulatory network that governs autophagy in response to inflammatory stimuli. The dual role of NF-κB—stimulating NLRP receptors while also activating mTOR, a known inhibitor of autophagy—underscores the intricate balance of signaling pathways that determine the fate of autophagic responses in inflammatory contexts. In the context of this study, where Gal-3 KO is associated with lower concentrations of p-NF-κB and increased autophagy, this suggests a potential regulatory role for Gal-3 in modulating these inflammatory signaling pathways and their impact on autophagy. Unraveling these molecular interactions could provide novel insights into the mechanisms underlying kidney injury and may offer avenues for targeted therapeutic interventions.

Our notion of TNF-mediated NF-κB activation further emphasizes the complexity of the relationship between inflammation, NF-κB, and autophagy. The dual mechanism by which TNF-mediated NF-κB activation suppresses autophagy, involving both reactive oxygen species’ (ROS) inhibition and mTOR activation, highlights the multiple levels at which these pathways can intersect and modulate cellular responses [36]. The suppression of autophagy by inhibiting ROS and activating mTOR in the context of TNF-mediated NF-κB activation suggests a concerted effort to downregulate cellular self-digestion during the experience of inflammatory conditions. This is in line with the concept that, in the presence of proinflammatory signals, cells may prioritize other processes over autophagy to deal with immediate stressors. Considering the intricate connections between NF-κB, TNF, and autophagy, our findings of altered NF-κB levels in Gal-3 KO mice and the associated changes in autophagy add valuable information to the broader understanding of these regulatory networks.

Wang et al. have also shown up-regulation of the β-catenin signaling pathway in AKI [37]. Our observations align with the notion that β-catenin activation is often considered a negative regulator of autophagy. This is grounded in the idea that high levels of autophagy, a process associated with cellular self-digestion and recycling, may be incompatible with the promotion of cell proliferation and survival, which are roles often attributed to β-catenin [37]. The relationship between β-catenin and autophagy highlights the intricate balance that cells maintain between processes related to cell growth and those related to cellular homeostasis and stress responses. The negative regulation of autophagy by β-catenin suggests a coordinated cellular strategy where the activation of certain pathways may suppress autophagy to prioritize other cellular functions. Our observation of alterations in β-catenin levels in Gal-3 KO mice adds to the complexity of the regulatory network involving autophagy and various signaling pathways. For instance, Thao et al. report that the activation of β-catenin inhibits the expression of Beclin-1, an important player in autophagic flux [38]. Conversely, it has been reported that autophagy activation was able to downregulate β-catenin signaling by degrading β-catenin [39]. 

Taking these findings together with the lower autophagic flux in CIS-treated Gal-3 wild-type mice than in CIS-treated Gal-3 KO mice in our study supports the view that the raised Gal-3 is associated with a lower autophagic flux. 

The reciprocal relationship between NF-κB and Gal-3, as indicated by studies showing NF-κB’s involvement in the induction of Gal-3 [40] and Gal-3’s role in the activation of NF-κB [41], underscores the complex regulatory cross-talk between these two signaling entities. This kind of bidirectional relationship often implies a feedback loop, where the activation of one component influences the activity of the other, creating a dynamic regulatory network. The identification of Gal-3 as a novel binding partner of β-catenin adds another layer to the complexity of the interactions between Gal-3 and signaling pathways related to cell proliferation and survival [42]. The involvement of Gal-3 in various cellular processes, including its associations with NF-κB and β-catenin, suggests a multifaceted role for Gal-3 in modulating cell functions and responses to stress or inflammation. Our study demonstrates changes in NF-κB and β-catenin levels in Gal-3 KO mice, and these complex relationships align with and contribute to our understanding of how Gal-3 may influence various signaling pathways in the context of kidney injury induced by CIS. 

These findings suggest that the role of Gal-3 in autophagy can also be mediated through the activation of NF-κB and Wnt/β-catenin pathways. Gal-3 appears to link the prosurvival signal transduction pathways which were investigated in our study. These pathways also have cross-talk between them at various levels. Wnt/β-catenin pathway players modulate many of their effects through interaction with NF-κB and, reciprocally, NF-κB also influences the Wnt/β-catenin signaling pathway [43]. The cross-talk between the Wnt/β-catenin and NF-κB signaling pathways adds depth to our understanding of the interconnection of these pathways. The reported interactions and reciprocal influences among Wnt/β-catenin and NF-κB further highlight the intricate regulatory network that governs cell survival, proliferation, and responses to stress. In this context, we have observed that Gal-3 plays a central connector role in these prosurvival signal transduction pathways and that their interaction with autophagy proteins is noteworthy. By influencing or being influenced by multiple signaling pathways, Gal-3 emerges as a key player in the coordination of cellular responses. The interplay between these pathways and the involvement of Gal-3 could have profound implications for conditions involving cellular stress, inflammation, and dysregulated autophagy, such as CIS-induced AKI. Understanding these complex interactions at the molecular level provides a foundation for identifying potential therapeutic targets that could modulate these pathways to manage kidney injury and improve patient outcomes.

## 4. Materials and Methods

The mouse model of AKI employed in our study has been thoroughly documented in the existing literature and widely adopted in laboratories globally for investigating AKI [44].

### 4.1. Mouse Model of Acute Tubular Necrosis

Male C57B6/J and B6.Cg-Lgals3 <tm 1 Poi>/J Gal-3 knockout (KO) mice weighing 25–30 g were utilized in this study. The mice were maintained on a standard diet and housed five per cage under a 12 h light and dark schedule for at least 1 week before CIS administration. All animal experimental procedures adhered to ethical guidelines and were approved by the United Arab Emirates University Animals Research Ethics Committee (Protocol: ERA_2019_6037). Freshly prepared CIS (Aldrich, Milwaukee, WI, USA) at a concentration of 0.5 mg/mL in sterile normal saline was administered intraperitoneally (IP) to the mice at a dosage of 25 mg/kg body weight [44]. Control mice received vehicle (normal saline) via the same route. Following CIS or vehicle administration, mice were allowed free access to food and water. The euthanasia procedure commenced with intraperitoneal injection of Phenobarbitone (70 mg/kg). Subsequently, kidneys were removed, and blood samples were collected 96 h after CIS administration. Blood was collected in EDTA vacutainers and centrifuged at 3000 RPM for 15 min, and plasma was aliquoted and stored at −80 °C until further analysis. Kidneys were washed in ice-cold phosphate-buffered saline (PBS). One half of each kidney was promptly frozen in liquid nitrogen and stored in a −80 °C freezer. The other half was fixed in 10% buffered formal saline for 24 h for subsequent analysis. This rigorous protocol ensured the ethical treatment of animals and the collection of reliable data for the study of AKI.

### 4.2. Sample Processing for Protein Extraction

Total protein extraction from kidney samples involved homogenization in lysis buffer, followed by centrifugation and collection of the supernatant. For total cell lysate, kidneys were thawed, weighed, and placed in cold lysis buffer comprising 50 mM Tris, 300 mM NaCl, 1 mM MgCl_2_, 3 mM EDTA, 20 mM β-glycerophosphate, 25 mM NaF, 1% Triton X-100, 10% *w*/*v* Glycerol, and a protease inhibitor tablet (Roche Complete protease inhibitor cocktail tablets). Homogenization was performed on ice using a homogenizer (IKA T25 Ultra Turrax, Staufen, Germany). The samples were then centrifuged at 14,000 RPM for 15 min at 4 °C, and the supernatant was collected, aliquoted, and stored at −80 °C for further analysis. Nuclear and cytoplasmic protein extraction followed a standard protocol. Briefly, kidney samples were thawed on ice, weighed, and homogenized on ice with a buffer containing Tris HCl 10 mmol/L, CaCl_2_ 3 mmol/L, MgCl_2_ 2 mmol/L, EDTA 0.1 mmol/L, Phenylmethanesulfonyl fluoride (PMSF) 0.5 mmol/L, Sucrose 0.32 mmol/L, Dithiothreitol (DTT) 1 mmol/L, Nonidet P-40 (NP-40) 0.5%, and Protease inhibitor cocktail 1%. The homogenates were centrifuged at 800 RPM for 10 min, and the supernatant was kept as the cytoplasmic fraction. The pellet was washed twice with homogenization buffer without NP-40 and resuspended with a low-salt buffer containing HEPES 20 mmol/L, MgCl_2_ 1.5 mmol/L, KCl 20 mmol/L, EDTA 0.2 mmol/L, Glycerol 25%, PMSF 0.5 mmol/L, and DTT 0.5 mmol/L. After incubation on ice for 5 min, an equal volume of high-salt buffer containing HEPES 20 mmol/L, MgCl_2_ 1.5 mmol/L, KCl 800 mmol/L, EDTA 0.2 mmol/L, Glycerol 25%, PMSF 0.5 mmol/L, DTT 0.5 mmol/L, NP-40 1%, and protease inhibitor cocktail 1% was added. The mixture was incubated on ice for 30 min then centrifuged at 14,000 RPM for 15 min at 4 °C, and the supernatants were saved as the nuclear fraction. The total protein concentration was determined using the BCA protein assay method (Thermo Scientific Pierce BCA Protein Assay Kit, Waltham, MA USA).

### 4.3. Sample Processing for Histology

The kidneys were excised, rinsed with ice-cold PBS, and weighed. Kidney sections were placed in cassettes and directly fixed in 10% buffered formalin. These sections underwent dehydration using increasing concentrations of ethanol, clearing with xylene, and embedding in paraffin. Three-micrometer sections were then prepared from the paraffin blocks and subjected to staining with hematoxylin and eosin as well as periodic acid Schiff (PAS) using standard procedures. The histopathologist involved in this project assessed and evaluated the stained sections.

#### 4.3.1. Immunofluorescent Labeling

Five-micrometer sections underwent deparaffinization with xylene and rehydration with graded alcohol. These sections were then subjected to retrieval solution with a high pH (pH 9) in a water bath at 95 °C for 20 min. Afterward, the sections were washed with distilled water for 5 min followed by PBST buffer for an additional 5 min. Subsequently, the sections were incubated with various primary antibodies: anti-Gal-3 antibody (Rabbit Polyclonal, 1:100, Santa Cruz Biotechnology, Dallas, TX, USA), anti-LC3B antibody (Mouse monoclonal, 1:100, Cell Signaling Technology, Danvers, MA, USA), anti-p62 antibody (Rabbit monoclonal, 1:100, Cell Signaling Technology, Danvers, MA, USA), anti-phospho mTOR antibody (Rabbit monoclonal, 1:100, Abcam, Cambridge, MA, USA), anti-p-AMPK antibody (Rabbit monoclonal, 1:100, Cell Signaling Technology, Danvers, MA, USA), anti-beta-catenin antibody (Rabbit Polyclonal, 1:100, Abcam, Cambridge, MA, USA), and anti-phospho-NF-κB antibody (Rabbit Polyclonal, 1:100, Abcam, Cambridge, MA, USA). This incubation took place overnight at room temperature. After washing with PBST, the sections were further incubated with donkey anti-rabbit Alexa Fluor 488 or donkey anti-mouse Rhodamine (Jackson Immune Research Laboratories, Bar Harbor, ME, USA, 1:100) antibodies. For double immunofluorescent labeling, sections were incubated overnight with two primary antibodies (mouse anti-LC3B and rabbit anti-p62 or mouse anti-LC3B and rabbit anti-Gal-3). Afterward, sections were washed with PBST and incubated with a combination of donkey anti-rabbit Alexa Fluor 488 and donkey anti-mouse Rhodamine antibodies. Following three washes with PBST, each lasting 5 min, the sections were mounted with DAPI-water-soluble mounting medium (Abcam, Cambridge, MA, USA) and observed using an Olympus Fluorescent microscope. Positive control sections were included in the procedure, and for negative controls, the primary antibody was omitted from sections, with the entire process carried out in the same manner as described above. Both positive and negative controls were utilized in every batch of stained slides. 

#### 4.3.2. Morphometric Analysis

Morphometric analysis of ATN was conducted by two investigators participating in this study. The analysis of p-AMPK, mTOR, phospho-NF-κB, and beta-catenin expression in renal tubular cells utilized ImageJ software version 1.54h December 2023 (http://rsbweb.nih.gov/ij/). P-AMPK, mTOR, phospho-NF-κB, and beta-catenin labeling were determined by counting the number of positive cells in randomly selected high-power fields (HPF) in the kidney. The mean numbers of positive cells were then converted from per-HPF to per-mm^2^ (each mm^2^ = 4 HPF). For p-AMPK and mTOR labeling, cells were considered positive with a cytoplasmic staining pattern. For phospho-NF-κB labeling, cells were considered positive with a nuclear and cytoplasmic staining pattern. For beta-catenin labeling, cells were considered positive with a cytoplasmic staining pattern.

### 4.4. Enzyme Linked Immunosorbent Assay

Kidney concentrations of Gal-3 and beta-catenin were determined using the DuoSet enzyme-linked immunosorbent assay (ELISA) Development kit from R&D Systems (Minneapolis, MN, USA). For LC3B, p-AMPK, mTOR, p62, Beclin-1, ATG13, Ulk-1, ATG5, ATG12, and ATG9A, kidney concentrations were determined using MyBioSource ELISA kits (San Diego, CA, USA) for sandwich ELISA, following the standard procedure according to the manufacturer’s instructions. Kidney concentrations of phospho-NF-κB for sandwich ELISA were determined using the Cell Signaling Technology ELISA kit (Danvers, MA, USA), also following the standard procedure according to the manufacturer’s instructions. Absorbance readings were taken at 450 nm using a 96-well plate spectrophotometer (BioTek EL × 800 microplate ELISA reader, Winooski, VT, USA). Concentrations of Gal-3, beta-catenin, phospho-NF-κB, LC3B, p-AMPK, mTOR, p62, Beclin-1, ULK-1, ATG13, ATG5, ATG12, and ATG9A in kidney samples were determined by interpolation from a standard curve. The measured levels were then normalized to total protein concentrations.

### 4.5. Plasma Samples Analysis

Plasma levels of urea and creatinine were measured using an automated analyzer Integra 400 Plus (Roche Diagnostics, Mannheim, Germany).

### 4.6. Statistical Analysis

All statistical analyses were conducted using GraphPad Prism Software version 5. Multiple comparisons between the various groups were performed using one-way analysis of variance (ANOVA), followed by Newman–Keuls post hoc multiple range tests. Comparisons between two groups were conducted using Student’s *t*-test. The data are presented as mean ± standard error (S.E.M), and P values less than 0.05 are considered statistically significant.

## 5. Conclusions

Our findings affirm the connection between Gal-3 and autophagy, revealing its central role as a connector with prosurvival signaling proteins. Gal-3 plays a pivotal role in orchestrating cellular responses by interacting with prosurvival signal pathways and engaging with autophagy proteins. Notably, our observations highlight that the absence of Gal-3 can enhance autophagy in CIS-induced ATN.

## Data Availability

The data presented in this study are available on request from the corresponding author.
